# Physical activity in young children and their parents–An Early STOPP Sweden–China comparison study

**DOI:** 10.1038/srep29595

**Published:** 2016-07-12

**Authors:** Elin Johansson, Hong Mei, Lijuan Xiu, Viktoria Svensson, Yueling Xiong, Claude Marcus, Jianduan Zhang, Maria Hagströmer

**Affiliations:** 1 Karolinska Institutet, Department of Clinical Science, Technology and Intervention, Division of Pediatrics, Stockholm, Sweden; 2 Huazhong University of Science and Technology, Tongji Medical College, School of Public Health, Department of Maternal and Child Health Care, Wuhan, P.R. China; 3 Karolinska Institutet, Department of Neurobiology, Care Sciences and Society, Division of Physiotherapy, Karolinska University Hospital, Department of Physical Therapy, Stockholm, Sweden

## Abstract

Understanding about socio-cultural differences in physical activity in children with high and low risk for obesity can help tailor intervention programs in different settings. This study aimed to compare objectively measured physical activity in two-year-olds and their parents, living in Stockholm, Sweden, and Wuhan, China. Data from Early STOPP was used. Children and parents wore an accelerometer in connection with the child’s second birthday. Weekly and hourly patterns were examined. Correlation between child and parental physical activity was assessed. Data on 146 Swedish and 79 Chinese children and their parents was available. Children, mothers and fathers in Stockholm were significantly more active than their counterparts in Wuhan (children; 2989 (SD 702) vs. 1997 (SD 899) counts per minute (CPM), mothers 2625 (SD 752) vs. 2042 (SD 821) CPM; fathers 2233 (SD 749) vs. 1588 (SD 754) CPM). Activity levels were similar over a week for children and parents within both countries. No parental-child correlations, except for a paternal-son correlation in Stockholm, were found. Children, mothers and fathers in Stockholm are more active compared with their counterparts in Wuhan. Interventions to increase physical activity needs to take cultural aspects into account, also when targeting very young children.

Physical activity is believed to influence health already during infancy^1^. Yet, little is known about physical activity levels and patterns among children under 3 years of age. It is a common assumption that young children have an endogenous drive to be active^2^ and it is difficult to affect their activity levels^3^. It does not seem like sex and BMI influence physical activity at this early age^4^. However, most studies of physical activity levels and patterns in children have been conducted in culturally similar countries like the US, Australia and Great Britain^5^,^6^,^7^. In many western countries, most two–year-old children attend preschool during weekdays, whereas in China, children taken care of by their grandparents is a norm^8^. Such differences likely affect the establishment of early health behaviors, such as physical activity patterns. Also, parental physical activity has been suggested as a possible correlate of child physical activity but this relationship needs further exploration^9^,^10^,^11^,^12^. The knowledge about how activity patterns differ in socio-culturally diverse countries and between children with high and low risk for obesity can help tailor interventions to promote physical activity at this early age in different settings.

Early Stockholm Obesity Prevention Project (Early STOPP) is an ongoing longitudinal study of children, 1–6 years of age, with high and low risk of becoming obese, based on parental weight status^13^. The study has two geographical sites, one in Stockholm, the largest city in Sweden, and one in Wuhan, one of the largest cities in China. The study protocols are identical and physical activity is assessed each year with accelerometry in children, mothers and fathers concurrently.

## Aim

The primary aim of this explorative cross-sectional study was to compare physical activity patterns between two-year-old children living in Stockholm and Wuhan, with high and low risk for obesity, based on parental Body Mass Index (BMI). A secondary aim was to compare parental physical activity levels by country and to assess correlations in parental-child activity.

## Methods

### Participants

Data from the two-year-old follow-up visit for families participating in the Early STOPP *Sweden* and *China* was used^13^. Baseline data collection in Stockholm, *Sweden*, started in 2009. Families (n = 199) with either one obese or two overweight parents with an infant were recruited from child health care centres before the child’s first birthday. These high-risk families were allocated to either intervention or control based on randomization of child health care centres. Since there were no differences in any physical activity variable between intervention and control families, they were merged and were referred to as the high-risk group (HR). A low-risk group (LR) of families with normal weight parents was also recruited (n = 59). Out of these, 199 families attended the follow-up visit.

Starting in 2010, families with a child under one year of age (n = 289) were recruited in Wuhan, *China*. The low-risk group was defined as families with either two normal weights or one normal weight and one overweight parent. In total, 202 families remained to follow-up but 123 families were excluded since the child did not have at least one day of physical activity data.

Early STOPP was approved by the Stockholm Regional Ethical Review Board (2009/217-31/2) and by the Ethical Committee (IORG0003571) of Tongji Medical College, Huazhong University of Science and Technology in China, and the methods were carried out in accordance with this approval. All families signed informed consent prior to their inclusion. The study has also been registered in Clinical Trials Registry (clinical trials.gov, ID: ES-2010).

### Anthropometric measurements

In Stockholm, families visited the research center within two months before or after the child’s second birthday. In Wuhan, home visit was conducted by two trained research assistants within two weeks from the child’s second birthday. Height was measured in both children and parents with a stadiometer to the nearest 0.1 cm and body weight was measured to the nearest 0.1 kg with a scale (Tanita HD-316, Tanita Corp.; Tokyo, Japan and Xiangshan EB9272H, Xiangshan Corp, Zhongshan City, China, in Stockholm and Wuhan, respectively). All measures were taken three times and the average height and weight were used to calculate BMI. Children were classified as normal weight, overweight or obese according to Cole *et al.*^14^. Swedish parents were classified as overweight if their BMI was ≥25 kg/m^2^ and as obese if their BMI was ≥30 kg/m^2^. Since Chinese people have a higher percentage of body fat compared with western people at the same BMI, 24 kg/m^2^ and 28 kg/m^2^ were used as BMI cut-offs for overweight and obesity in Chinese parents^15^.

### Measurement of physical activity

In both countries physical activity was measured with accelerometry during the week before or after the two-year visit. Both parents and children were measured concurrently. The Actigraph GT3X+ accelerometer was worn around the non-dominant wrist^16^,^17^, determined by the parents, for 24 hours during seven consecutive days. Data was sampled at 30 Hz and, as in previous studies, summed in 5 second epochs for children^18^ and in 60 second epochs for parents^19^. A shorter epoch length has been recommended for children in order to capture their intermittent activity pattern with short bursts of activity at a high intensity^4^,^20^,^21^. ActiLife software, version 6.9.2 was used for initialization, downloading and analysis of data. Data on average physical activity, expressed as counts per minute (CPM) was extracted for the vector magnitude (VM), which is a measure of the three axis combined 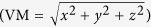. The VM has been found to give a better estimate of physical activity than the vertical axis (VA) alone^22^. Means per day and means per hour were calculated. For children, age and placement site specific intensity thresholds of the VM, developed by Johansson *et al.*^23^ was used to assess time spent in sedentary behavior, low- and high intensity physical activity. To exclude sleep time, the hours between 9:00 pm and 6:00 am was removed for children^24^ and the hours between 10:00 pm and 6:00 am for parents, based on estimated average bed time from sleep records. Days with less than 15 and 16 hours of recording were considered invalid for children and parents respectively and were therefore deleted. Further, days with VM CPM <100 were also excluded, since it is unlikely that the monitor could have been worn such days.

### Potential confounders

In both countries, parents answered questionnaires in connection with the two-year visit. Data on birth weight and length, number of siblings and the siblings ages, pregnancy length (weeks), at what age the child started to walk (months), type of child care (preschool/parents/grandparents/others) and parental education (families where at least one parent had at least 12 years of school were considered highly educated) was extracted.

### Statistical analysis

Descriptive data are presented as mean (SD) or N (%). Comparisons between countries and risk groups were assessed with t-test (continuous variables) and Chi-square test (categorical variables). Differences in all measured variables were compared between those with one vs those with two and those with at least four days of valid accelerometer data. Since no differences were found, all children, mothers and fathers with at least one day of accelerometer data were included. The Kolmogorov-Smirnov test was used to exam the normality of the distribution. The average physical activity (expressed as VM CPM) for both parents and children was found to be normally distributed as well as the amount of time in high intensity for children. For children, time being sedentary was negatively skewed and time in low intensity activity was positively skewed.

Average physical activity, in total, by country and by risk group, was calculated for children and parents. Time (minutes) spent in sedentary behavior, low- and high intensity physical activity was presented for children.

To assess differences between weekdays and weekends in average physical activity (parents and children) and time in different intensities (children), t-test was used. Skewed data was log-transformed prior to statistical analysis.

Mean CPM and standard deviations were plotted graphically to demonstrate the hourly pattern. Differences in the hourly pattern between children in Stockholm and Wuhan were assessed with generalized estimating equations models.

Mother-boy, mother-girl and father-boy and father-girl associations in average physical activity during weekdays and weekend days were examined using partial correlation analysis, adjusting for the potential confounders.

All analysis was performed in SPSS for Windows, version 22 (SPSS Inc., Chicago, IL). Significance level of 0.05 was used.

## Results

### Descriptive characteristics

Descriptive characteristics of included children and parents are shown in Tables 1 and 2, respectively. Data on 146 children, 145 mothers and 140 fathers was available in the Swedish cohort. In the Chinese cohort the corresponding number was 79 children, 69 mothers and 61 fathers, respectively.

Both Swedish and Chinese children had a mean BMI of 16.9 kg/m^2^, and 14% and 12% respectively were considered overweight/obese in the two countries. Swedish parents were significantly older, taller and had a higher BMI compared with their Chinese counterparts (p < 0.05).

Swedish children and parents had a mean number of valid days of 6.7 (SD 0.7 for children and 0.9 for parents). Chinese children had 4.8 (SD 2.2), mothers 5.4 (SD 1.8) and fathers 5.6 (SD 1.7) valid days. The number of valid days differed significantly for both children and parents by county (p < 0.05).

### Physical activity among children in Stockholm and Wuhan

**Figure 1:**
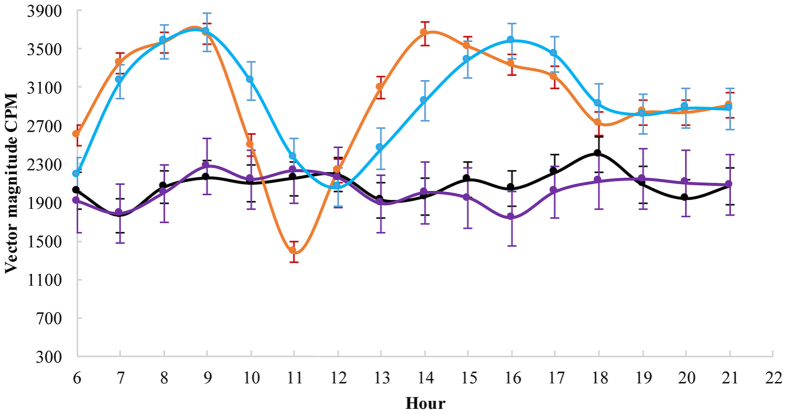
The hourly average physical activity in Swedish and Chinese children on weekdays and weekends. Mean CPM (95% CI). 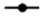 China weekday 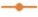 Sweden weekday 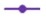 China weekday 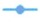 Sweden weekday.

Table 2 displays descriptive data on child physical activity by country, for the total sample and by risk group. In comparison with Swedish children, Chinese children had significantly lower average physical activity levels and spent more time being sedentary and less time at low and high intensity physical activity (p < 0.05). Figure 1 shows the hourly pattern of average activity in Swedish and Chinese children on weekdays and weekend days. No significant differences in physical activity patterns were seen between weekdays and weekends in children. Activity levels differed significantly between the countries at all times during the day, except for at 12 am.

### Physical activity among children with high and low risk for obesity

Chinese children in the HR group spent less time being sedentary and more time in low and high intensity physical activity compared with children in the LR group (p < 0.05) (Table 2). No difference was seen between risk-groups in the Swedish cohort.

### Parental physical activity

Swedish mothers and fathers were significantly more active than their counterparts in China (p < 0.05) and fathers were significantly less active compared with mothers, in both countries (p < 0.001) (Table 3). There were no differences in average physical activity between risk-groups in either country. No significant differences in physical activity patterns were seen between weekdays and weekends in parents in either country.

### Parent – child associations

**Figure 2:**
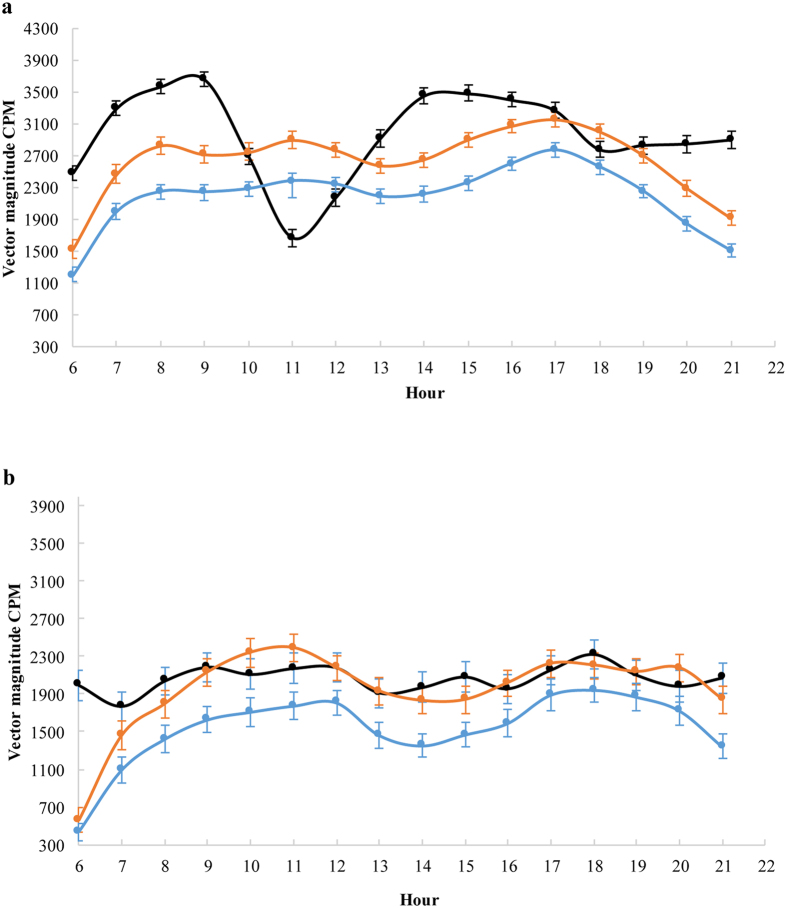
The hourly average physical activity in children, mothers and fathers in Sweden (**a**) and in China (**b**). Mean CPM (95% CI). 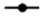 Children 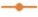 Mothers 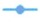 Fathers.

Figure 2a,b show the hour-by-hour activity patterns of children, mothers and fathers in Sweden and China, respectively. In Sweden, fathers’ physical activity was positively correlated to boys’ activity on weekdays (p < 0.05, r = 0.49) and on weekends (p < 0.05, r = 0.37), adjusted for potential confounders. The physical activity of fathers and girls’ was not correlated, and maternal activity was not correlated with either boys’ or girls’ activity. Neither Chinese mother’s nor father’s activity was correlated to the child’s physical activity on either weekdays or weekends.

## Discussion

The main finding is that children, mothers and fathers in Stockholm are significantly more active than their counterparts in Wuhan. Parental and child physical activity are not associated, except for a positive association between Swedish fathers and sons.

One possible explanation to the identified difference in activity level between children in the two countries is difference in the type of day care. Almost all Swedish children aged 2–5 years attend preschool during weekdays. Swedish pre-schoolers have abundant of opportunities to play and to be physically active together with peers and preschool attendance has been positively associated with physical activity in 4–6-year olds^25^,^26^. The Swedish National Agency for Education has developed policy documents stating that the preschool should provide opportunities for the children to develop motor skills and coordination^27^. At least one part of the day is spent outdoors, which has been associated with increased physical activity in preschool children^10^. Lunch is most often served between 11 and 12 am and is followed by at least one hour of rest/sleep, explaining the low levels between 10 am and 1 pm. This pattern, with a decrease in activity around noon has been shown in 19 months old and 3–4 years olds in Australia and the US^5^,^6^,^28^. It can be hypothesised that the finding of a similar physical activity pattern during weekends is due to families trying to keep the same schedule of meal and rest during weekends as during weekdays. Keeping regular food and sleep habits are often encouraged at Swedish child health care centres. In Sweden, fathers are often highly involved in taking care of the child and engaging in child activities. According to the results from a previous meta-analysis, paternal activity, in comparison with maternal activity, was found to be more strongly correlated with child activity^29^. It has been found that boys generally receive more support to be physically active than girls^9^. It is possible that at this early age, fathers are particularly more engaged in their sons than their daughter’s physical activity.

In China, children usually stay at home until three years of age, and are often taken care of by grandparents living in the same household as the children, or in close proximity. Although nuclear family is the main construct in current China, extended family households (with three or more generations) still constitute a large proportion^30^, and it is a traditional, common and acceptable idea that grandparents involve in childcare, especially in early childhood^31^. Due to the one-child policy enacted in late 1970s, most families in urban China have one child only. Therefore both parents and grandparents tend to be overprotective of the only child and encourage less vigorous or even sedentary indoor activities as the outdoor environment and vigorous activities are often perceived as unsafe^32^. Grandparents engage heavily in taking care of their grandchildren in China. Therefore, they play a vital role in forming Chinese children’s dietary habits^8^ and likely influence physical activity as well. The substantial involvement of grandparents might at least partially explain the lack of parent-child association in physical activity in the Chinese participants.

In line with previous reviews, BMI was not associated with physical activity in young children^10^,^33^. Interestingly, we found that children with overweight or obese parents in Wuhan spent less time being sedentary and more time being physically active than children with normal weight parents. One possible explanation is that the parents in the high-risk group might be aware of the importance of physical activity and tend to encourage their children to be more physically active. In China, obesity is more common among adults, particularly men, with high educational level and income^34^,^35^. No differences in physical activity were found between children in the two risk-groups in the Swedish cohort, which is in accordance with previous studies on 2–5-year-olds living in western countries^5^,^10^. This finding, together with the differences in physical activity levels and patterns, indicates that socio-cultural context might play an important role in shaping physical activity behaviours even in early childhood.

Parents in Wuhan were significantly less active compared with the parents in Stockholm. During the past three decades China has experienced rapid urbanization^36^, which has led to dramatic changes in people’s life styles, such as increased occupational sedentariness and less active transportation^37^. Data from China Health and Nutrition Survey (CHNS) showed that between 1991 and 2006, average weekly physical activity among Chinese adults decreased by 32%, with reduction in occupational activity, domestic activity and transportation activity^38^. These changes are probably due to technological advancements in both work and living environment, improvement in public transportation as well as increasing use of private motorized vehicles^38^. It was reported that the Chinese males are engaging in less occupational and domestic activities compared with their female counterparts, which could explain the difference between mothers and fathers in this study^37^. A cross-national study showed that the odds of engaging in leisure time physical activity increased markedly with age, with the lowest level in people aged 18–35 years^39^, primarily due to heavy work load or working overtime, which are quite common in China^40^. It is also common for Chinese adults to have a nap after lunch, which could explain the decrease in activity between 1.00 and 4.00 pm. Another possible reason to the observed differences between the countries is related to the air quality. Many larger cities in China suffer from air-pollution, making outdoor activities difficult and perceived as unhealthy^41^. The air-pollution becomes worse in the afternoon and those hours are often spent indoors.

In summary, our findings indicate a need for promotion of physical activity among children and adults in urban China. It seems like endogenous drives for physical activity can be markedly modified by socio-cultural factors already as early as at age two. Increasing awareness among parents and grandparents, facilitate and encourage outdoor activities are possible keys to increase physical activity among children but there is a great need for more studies exploring this field of research.

In Stockholm, paternal physical activity was significantly correlated with boy’s physical activity on both weekdays and weekends. This is in accordance with previous studies which have shown that paternal activity has a stronger association with children’s activity than maternal activity^42^,^43^. In China, parental physical activity was not correlated with either boys or girls activity. This could be explained by that children spent most time together with grandparents. Data on grandparental physical activity was not available for this study but future studies should investigate their influence of grandparents in forming healthy habits for young children.

### Strengths and limitations

The major strength is the use of identical study protocol in both study sites. Physical activity and other key variables like weight and height was measured objectively and the same procedures for data cleaning and analysis were used in both countries. Within the families, parent’s and children’s physical activity were measured during the same week. Seasonal variations should not have affected the results since data was collected around the year.

Some limitations need to be addressed. Families in Wuhan had only half as many days with available physical activity data as families in Stockholm, which could have affected the results. Average physical activity was used as outcome measure in the parents. It is a gross measure but the lack of intensity cut-offs for wrist-worn Actigraphs or standardized ways of performing pattern recognition of raw data made it unsuitable to use any other measure. Nevertheless, total or average counts have been suggested as a valuable measure^44^. By using accelerometry, activities like carrying heavy loads and bicycling, are underestimated. It is possible that parents in Wuhan were doing such activities more frequently than their peers in Stockholm, which could partially explain their lower average activity levels. Future studies should gather contextual information about activities performed. Such information would also increase the knowledge about parental-child co-participation in activities. Daytime naps were not differentiated from sedentary time, which is why sedentary time likely was over-estimated. The included families were living in large cities, had a higher level of education compared with the general populations and were recruited based on parental BMI. The disparities between rural and urban areas in the prevalence of obesity are significant^45^,^46^, therefore results from this study should be generalized with caution.

## Conclusion

This study shows the major differences in physical activity patterns between two-year-old children in Stockholm and Wuhan. Children and their parents in Wuhan are less active and have a different physical activity pattern compared with their counterparts in Stockholm. There seems to be no parental-child association in physical activity, except for in Stockholm where a paternal-son association was found. Interventions to increase physical activity needs to take cultural aspects into account, already at this young age.

## Additional Information

**How to cite this article**: Johansson, E. *et al.* Physical activity in young children and their parents–An Early STOPP Sweden–China comparison study. *Sci. Rep.* **6**, 29595; doi: 10.1038/srep29595 (2016).

## Figures and Tables

**Table 1:** Child descriptive characteristics.

	Sweden		China	
	Total (N = 146)	Missing (n)	Total (N = 79)	Missing (n)
Age (years), mean (SD)	2.03 (0.1)		2.04 (0.13)	
Male, n (%)	75 (51)		44 (56)	
Birth weight (g), mean (SD)	3634 (558)*	6	3464 (548)	2
Birth length (cm), mean (SD)	50.6 (2.5)	4	50.6 (1.9)	1
Weight (kg), mean (SD)	13.1 (1.6)*		13.6 (1.7)	
Length (cm), mean (SD)	87.9 (3.2)*		89.6 (3.4)	
BMI (kg/m^2^), mean (SD)	16.9 (1.4)		16.9 (1.7)	
BMI category, n (%)				
Normal weight	126 (86)		69 (88)	
Overweight	17 (12)		5 (6)	
Obesity	3 (2)		5 (6)	
Child first born, n (%)	60 (42)*	2	64 (82)	1
Child care, n (%)		4		6
Preschool	138 (97)*			
Parents			13 (18)*	
Grandparents			53 (73)*	
Others	4 (3)*		7 (9)	
Started walking (months), mean (SD)	12.0 (2.0)*	11	12.7 (2.3)	6
Pregnancy weeks, mean (SD)	39.0 (4.3)	11	39.0 (1.3)	2
Family risk group, n (%)				
High-risk	107 (73)*		41 (52)	
Low-risk	39 (27)*		38 (48)	

^*^Significant difference (p < 0.05) between Sweden and China.

**Table 2:** Description of child physical activity.

	Sweden			China		
	Total (N = 146)	HR (N = 107)	LR (N = 39)	Total (N = 79)	HR (N = 41)	LR (N = 38)
Valid days	6.7 (0.7)#	6.7 (0.7)	6.7 (0.9)	4.8 (2.2)	4.9 (2.2)	4.7 (2.2)
VM CPM	2989 (702)#	2991 (700)	2984 (709)	1997 (899)	2106 (868)*	1875 (918)
Sedentary (min/day)	445 (68)#	445 (67)	444 (71)	545 (99)	533 (94)*	558 (103)
Low PA (min/day)	261 (49)#	261 (47)	262 (53)	195 (79)	204 (74)*	185 (84)
High PA (min/day)	73 (29)#	74 (29)	72 (28)	40 (26)	43 (27)*	37 (25)

Mean (SD).

^#^Significant difference (p < 0.05) between Sweden and China.

^*^Significant difference (p < 0.05) between HR and LR within country.

CPM = counts per minute; VM = vector magnitude.

**Table 3:** Parental descriptive characteristics.

	Sweden				China			
	Mother		Father		Mother		Father	
	Total (N = 145)	Missing (n)	Total (N = 140)	Missing (n)	Total (N = 69)	Missing (n)	Total (N = 61)	Missing (n)
Age (years), mean (SD)	34.8 (4.7)*		37.2 (5.5)*	6	29.1 (3.6)	1	31.6 (4.8)	2
Weight (kg), mean (SD)	82.3 (20.0)*	1	89.4 (15.8)*	6	60.8 (10.1)		79.0 (11.6)	1
Height (cm), mean (SD)	167.0 (7.1)*	2	180.4 (6.4)*	7	160.0 (5.1)		180.4 (6.4)	1
BMI (kg/m^2^), mean (SD)	29.5 (7.0)*	2	27.6 (4.8)*	11	23.9 (3.6)		26.4 (3.6)	1
Educational level, n (%)		5		4		1		1
High	105 (75)		102 (75)		49 (72)		46 (77)	
Low	35 (25)		34 (25)		19 (28)		14 (23)	
Valid days,mean (SD)	6.7 (0.9)*		6.7 (0.9)*		5.4 (1.8)		5.6 (1.7)	
VM CPM,mean (SD)	2625 (752)*		2233 (749)*		2042 (821)		1588 (754)	

^*^Significant difference (p < 0.05) between Sweden and China.
